# Monomer-Induced Customization of UV-Cured Atelocollagen Hydrogel Networks

**DOI:** 10.3389/fchem.2018.00626

**Published:** 2018-12-17

**Authors:** He Liang, Stephen J. Russell, David J. Wood, Giuseppe Tronci

**Affiliations:** ^1^Clothworkers' Centre for Textile Materials Innovation for Healthcare, School of Design, University of Leeds, Leeds, United Kingdom; ^2^Biomaterials and Tissue Engineering Research Group, School of Dentistry, St. James's University Hospital, University of Leeds, Leeds, United Kingdom

**Keywords:** type I atelocollagen, covalent network, UV-curing, monomer, 4-vinylbenzyl chloride, methacrylic anhydride

## Abstract

The covalent functionalization of type I atelocollagen with either 4-vinylbenzyl or methacrylamide residues is presented as a simple synthetic strategy to achieve customizable, cell-friendly UV-cured hydrogel networks with widespread clinical applicability. Molecular parameters, i.e., the type of monomer, degree of atelocollagen functionalization and UV-curing solution, have been systematically varied and their effect on gelation kinetics, swelling behavior, elastic properties, and enzymatic degradability investigated. UV-cured hydrogel networks deriving from atelocollagen precursors functionalized with equivalent molar content of 4-vinylbenzyl (*F*_4*VBC*_ = 18 ± 1 mol.%) and methacrylamide (*F*_*MA*_ = 19 ± 2 mol.%) adducts proved to display remarkably-different swelling ratio (*SR* = 1963 ± 58–5202 ± 401 wt.%), storage modulus (*G*′ = 17 ± 3–390 ± 99 Pa) and collagenase resistance (μ_*rel*_ = 18 ± 5–56 ± 5 wt.%), similarly to the case of UV-cured hydrogel networks obtained with the same type of methacrylamide adduct, but varied degree of functionalization (*F*_*MA*_ = 19 ± 2 – 88 ± 1 mol.%). UV-induced network formation of 4VBC-functionalized atelocollagen molecules yielded hydrogels with increased stiffness and enzymatic stability, attributed to the molecular rigidity of resulting aromatized crosslinking segment, whilst no toxic response was observed with osteosarcoma G292 cells. Although to a lesser extent, the pH of the UV-curing solution also proved to affect macroscopic hydrogel properties, likely due to the altered organization of atelocollagen molecules during network formation. By leveraging the knowledge gained with classic synthetic networks, this study highlights how the type of monomer can be conveniently exploited to realize customizable atelocollagen hydrogels for personalized medicine, whereby the structure-property relationships can be controlled to meet the requirements of unmet clinical applications.

## Introduction

As a simple mimetic of the extracellular matrix (ECM) of biological tissues hydrogels have been widely applied in the biomedical field (Zhang and Khademhosseini, 2017), for applications including regenerative medicine (Heller et al., 2013), wound care (Konieczynska et al., 2016), and controlled drug delivery (Chen et al., 2017). The high swelling in aqueous medium makes hydrogels inherently soft, so that molecular manipulation of their constituent building blocks (Nimmo and Shoichet, 2011) is key to enable customized properties and functions for personalized medicine.

At the molecular scale, hydrogels typically consist of hydrophilic polymer networks, crosslinked via either physical or covalent linkages, whilst short amino acidic sequences have also been proven to generate water-swollen supramolecular structures (Koch et al., 2018). Although various synthetic polymers have been employed (Aggeli et al., 2003; Fairbanks et al., 2009; Bulman et al., 2015; Qin et al., 2015, 2018; Sridhar et al., 2015; Gharaei et al., 2016), a great deal of attention has recently been paid to the use of natural, ECM-extracted, biopolymers as building blocks of multifunctional hydrogels (Van Vlierberghe et al., 2011; Head et al., 2016). The selection of biopolymer-based building blocks is attractive since it ensures that the resulting material has biomimetic features from the molecular up to the macroscopic scale, due to the presence of cell-binding sequences, fiber-forming biopolymer capability (with resulting fiber dimensions comparable to the ones found in the ECM) (Younesi et al., 2014), and the water-swollen macroscopic state, respectively. In comparison to synthetic polymers, however, ensuring customization and reliable structure-property relationships in biopolymer-based systems is highly challenging, due to the inherent batch-to-batch variability and the hardly-controllable secondary interactions between biopolymer chains (Salchert et al., 2004; Yang et al., 2016). Leveraging the knowledge gained with classic polymers, the synthesis of covalently-crosslinked hydrogel networks made of linear biopolymers, e.g., hyaluronic acid (Burdick and Prestwich, 2011), has been successful aiming to achieve defined structure-property relationships (Tronci et al., 2010). Other than linear biopolymers, such a level of customization is still hardly realized when employing biopolymers with increased organizational complexity, such as collagen, because of the inherently-limited chemical accessibility and solubility. Customization of these building blocks would on the other hand enable the formation of systems with superior biofunctionality and widespread clinical applicability. Type I collagen is one the most abundant structural proteins of connective tissues, and it constitutes the main organic component of bone, skin and tendon (Grant et al., 2009). As the most abundant collagen in humans, type I collagen-based hydrogels have been widely employed for therapeutics and diagnostics, with multiple commercial products being routinely used in the clinic (Neel et al., 2013). Other than its hierarchical, water-insoluble organization found in biological tissues, collagen *ex vivo* is predominantly extracted in the form of a water-soluble triple helix, due to the extraction-induced breakdown of covalent crosslinks of collagen found *in vivo*. To enable applicability of the extracted water-soluble product in physiological conditions, restoration of *in vivo*-like covalent crosslinks *ex vivo* is a promising strategy. Reaction of side chain terminations with bifunctional segments, e.g., diisocyanates (Olde Damink et al., 1995a; Kishan et al., 2015), diacids (Duan et al., 2014; Tronci et al., 2015b), and dialdehydes (Olde Damink et al., 1995b; Haugh et al., 2011), or carbodiimide-induced intramolecular crosslinking (Everaerts et al., 2008; Yunoki and Matsuda, 2008) proved to induce some adjustment in the mechanical properties of resulting collagen materials. At the same time, the use of toxic reagents and the relatively-long reaction time prevent the application of these synthetic strategies for the delivery of cell-friendly, *in-situ* forming hydrogels (Lee et al., 2013) or to achieve material customization at the bed side. Another challenge associated with above-mentioned strategies is that the enzymatic degradability of resulting collagen materials is still relatively quick, so that the requirements of specific clinical application, e.g., in guided bone regeneration, cannot be entirely fulfilled (Calciolari et al., 2018). Ultimately, above-mentioned crosslinking strategies are often associated with unwanted side reactions, so that defined structure-property relationships are hardly-developed (Yunoki and Matsuda, 2008; Tronci et al., 2010; Duan et al., 2014).

Rather than one-step crosslinking reactions, photoinduced network formations mechanisms have been recently investigated for the development of *in situ*-forming, cell-encapsulating collagen hydrogels (Brinkman et al., 2003). Derivatization of collagen triple helices with photoactive, e.g., methacrylamide, groups has been widely demonstrated to rapidly generate covalent networks via photoinduced free radical crosslinking (Gaudet and Shreiber, 2012) and UV-induced thiol-ene (Holmes et al., 2017) click reactions. Although resulting systems proved to display varied storage modulus (Gaudet and Shreiber, 2012; Ravichandran et al., 2016), collagen hydrogel customization often relies on the incorporation of a synthetic copolymer, e.g., polyethylene glycol (PEG), in the crosslinked network. Whist this approach affords wide tailoring in macroscopic properties, incorporation of the synthetic phase may affect the biofunctionality of resulting system. In an effort to expand the customizability of collagen hydrogels and avoid the use of copolymers, functionalization of collagen with 4-vinylbenzyl residues has recently been reported. UV-cured 4VBC-functionalized collagen triple helices proved to display significantly increased compression properties with respect to methacrylated variants (Tronci et al., 2016a), whereby the introduction of 4VBC aromatic rings was found to impact on the activity of matrix metalloproteinases (MMPs) *in vitro* (Tronci et al., 2016b; Liang et al., 2018). Although insightful, these studies did not systematically investigate the effect of the type of monomer and respective degree of functionalization on the macroscopic properties of resulting hydrogels, due to the limited chemical accessibility of the protein backbone. On the other hand, systematic investigations on the effect of covalently-coupled monomer and respective degree of functionalization on network properties could open up new avenues aiming to develop simple synthetic routes yielding customizable collagen systems for personalized medicine.

This study therefore is focused on the synthesis of photoactive atelocollagen precursors and consequent UV-cured networks, whereby the effects of (i) type of monomer, (ii) the degree of atelocollagen functionalization, and (iii) UV-curing aqueous solution were addressed. Pepsin-solubilized type I telopeptide-free collagen, i.e., atelocollagen, was selected as a purified, minimally-antigenic building block with comparable chemical composition, and dichroic properties with respect to acid-extracted type I collagen (Lynn et al., 2004; Tronci et al., 2016b). Firstly, photoactive atelocollagen precursors with comparable molar content, but different type of photoactive adduct, i.e., either 4-vinylbenzyl or methacrylamide adduct, were considered. Secondly, photoactive atelocollagen precursors with varied molar content of the same type of photoactive, i.e., methacrylamide, adduct were addressed. Ultimately, the aqueous solution employed for the solubilization of functionalized atelocollagen, i.e., hydrochloric acid, acetic acid and phosphate buffered saline solution, was also varied during network formation to investigate the effect of environmental conditions on resulting hydrogels. Hydrogel networks were prepared via UV-induced free radical crosslinking of photoactive atelocollagen precursors in the presence of 2-hydroxy-1-[4-(2-hydroxyethoxy) phenyl]-2-methyl-1-propanone (I2959), which was used as a water-soluble, cell-friendly photoinitiator (Holmes et al., 2017). Resulting UV-cured hydrogel networks were characterized with regards to their gelation kinetics, swelling and compression properties, enzymatic degradability, and cytotoxicity, aiming to establish defined structure-property relationships, and systematic material customization.

## Materials and Methods

### Materials

Pepsin-extracted type I bovine atelocollagen (AC, 6 mg·mL^−1^) solutions in 10 mM hydrochloric acid (HCl) were purchased from Collagen Solutions PLC (Glasgow, UK). 4-vinylbenzyl chloride (4VBC), methacrylic anhydride (MA), and triethylamine (TEA) were purchased from Sigma-Aldrich. I2959 and deuterium oxide were purchased from Fluorochem Limited (Glossop, UK). Ninhydrin 99% was supplied by Alfa-Aesar (Massachusetts, USA). Polysorbate 20, absolute ethanol and diethyl ether were purchased from VWR internationals. All other chemicals were purchased from Sigma-Aldrich unless specified.

### Synthesis of Photoactive Precursors

To achieve the MA-functionalized products, AC solutions (6 mg·mL^−1^ in 10 mM HCl) were diluted to a concentration of 3 mg·mL^−1^ via addition of 10 mM HCl and equilibrated to pH 7.5. MA and TEA were added at varied molar ratios with respect to the molar lysine content in AC ([MA][Lys]^−1^ = 0.1–25). When an MA/Lys molar ratio of 0.1–1 was selected, TEA was added with a 10 molar ratio with respect to the collagen lysine content. When an MA/Lys molar ratio of 25 was selected, an equimolar monomer content of TEA was added ([TEA] = [MA]). After 24 h, the functionalization reaction was stopped by precipitating the reacting mixture in 10-volume excess of absolute ethanol. Following at least 8-h incubation in ethanol, the reacted, ethanol-precipitated product was recovered by centrifugation and air dried. The 4VBC-functionalized AC was prepared following the above protocol, whereby polysorbate 20 (PS-20) was added prior to the functionalization reaction in order to mediate the solubility of 4VBC in water. Hence, the diluted (3 mg·mL^−1^) and pH-equilibrated AC solution was supplemented with 1 wt.% PS−20 (with respect to the weight of the diluted AC solution) prior to addition of 4VBC and TEA at a fixed molar ratio of 25 ([4VBC]·[Lys]^−1^ = 25; [4VBC] = [TEA]).

### Characterization of Reacted AC Products

TNBS assay (*n* = 3) was used to measure the derivatization of amino to vinyl groups and respective degree of AC functionalization, as previously reported (Tronci et al., 2016b; Liang et al., 2018). Briefly, 11 mg of dry samples was mixed with 1 mL of 4 wt.% NaHCO_3_ and 1 mL of 0.5 wt.% TNBS solution. The mixture was reacted at 40°C for 4 h, followed by addition of 3 mL of 6 N HCl for one more hour to induce complete sample dissolution. The solution was then cooled down to room temperature, diluted with 5 mL of distilled water, and extracted (×3) with 15 mL diethyl ether to remove any non-reacted TNBS species. An aliquot of 5 mL was collected and diluted in 15 mL of distilled water and the reading was recorded using an UV-Vis spectrophotometer (Model 6305, Jenway) against the blank. The molar content of primary free amino groups (largely attributed to the side chains of lysine) was calculated via Equation 1:

(1) $$\begin{array}{l} \frac{mol\left(Lys\right)}{g\left(AC\right)}=\frac{2\times Abs\left(346\;nm\right)\times 0.02}{1.46\times 10^{4}\times b\times x} \end{array}$$

where *Abs* (346 *nm*) is the UV absorbance value recorded at 346 nm, *2* is the dilution factor, *0.02* is the volume of the sample solution (in liters), 1.46 × 10^4^ is the molar absorption coefficient for 2,4,6-trinitrophenyl lysine (in M^−1^ cm^−1^), *b* is the cell path length (1 cm), and *x* is the weight of the dry sample. The degree of functionalization (*F*) was calculated via Equation 2:

(2) $$\begin{array}{l} F=\left(1-\frac{{mol\left(Lys\right)}_{funct.}}{{mol\left(Lys\right)}_{AC}}\right)\times 100 \end{array}$$

where *mol(Lys)*_*AC*_ and *mol(Lys)*_*funct*._represent the total molar content of free amino groups in native and functionalized atelocollagen, respectively.

Further confirmation of AC functionalization was assessed via Ninhydrin assay (*n* = 3). Briefly, 10 mg of the dry sample was mixed with 4 mL of distilled water and 1 mL of 8 wt.% Ninhydrin solution in acetone. The mixture was reacted at 100°C for 15 min and the reaction terminated by cooling in ice and adding 1 mL of 50% ethanol (v/v). The molar content of amino groups was measured by reading the absorbance at 570 nm against the blank. A standard curve calibration was prepared by measuring solutions containing known amount of atelocollagen.

TNBS and ninhydrin assays were ultimately coupled with ^1^H-NMR spectroscopy (JEOL ECA, 600 MHz). ^1^H-NMR spectra of native, MA- and 4VBC-functionalized AC were recorded following dissolution of 5 mg of dry sample in 1 mL of 10 mM deuterium chloride.

### Synthesis of UV-Cured AC Networks

Either MA- or 4VBC-functionalized AC products were dissolved at a fixed concentration of 0.8 wt.% in either 10 mM HCl (pH 2.1), 17.4 mM acetic acid (AcOH, pH 3.4) or 10 mM phosphate buffered saline (PBS, pH 7.5) solutions supplemented with 1 wt.% I2959 photo-initiator. Resulting AC solutions (0.8 wt.% functionalized AC, 0.992 wt.% I2959) were centrifuged at 3000 rpm for 5 min to remove any air bubble and then cast onto a 24 well plate (Corning Costar, 0.8 g per well). Well plates were irradiated with a UV lamp (346 nm, 8 mW cm^−2^, Spectroline) for 30 min on both top and bottom side, leading to the formation of hydrogels. Irradiation intensities were measured with an International Light IL1400A radiometer equipped with a broadband silicon detector (model SEL033), a 10 × attenuation neutral density filter (model QNDS1), and a quartz diffuser (model W). The UV-cured hydrogels were carefully removed from the plate and washed in distilled water (15 min, × 3), followed by dehydration *via* an ascending series of ethanol and air drying.

### Quantification of Swelling Ratio and Gel Content

Dry UV-cured samples (*n* = 4) of known mass (*m*_*d*_) were individually incubated in PBS (10 mM, pH 7.5, 1.5 mL) at room temperature for 24 h. The swelling ratio (*SR)* was calculated by Equation 3:

(3) $$\begin{array}{l} SR=\frac{m_{s}-m_{d}}{m_{s}}\times 100 \end{array}$$

where *m*_*s*_ is the mass of the PBS-equilibrated UV-cured sample.

The gel content (*n* = 4) was measured to investigate the overall portion of the covalent hydrogel network insoluble in 17.4 mM acetic acid solution (Liang et al., 2018). Dry collagen networks (*m*_*d*_: 10 mg−20 mg) were individually incubated in 2 mL of 17.4 mM AcOH for 24 h. Resulting samples were further air dried and weighed. The gel content (*G*) was calculated by Equation 4:

(4) $$\begin{array}{l} G=\frac{m_{1}}{m_{d}}\times \;100 \end{array}$$

where *m*_1_ is the dry mass of collected samples.

### Compression Test

PBS-equilibrated hydrogel discs (Ø: 14 mm; *h*: 5–6 mm, *n* = 3) were compressed at room temperature with a compression rate of 3 mm·min^−1^ (Instron ElectroPuls E3000). A 250 N load cell was operated up to complete sample compression. Stress-compression curves were recorded and the compression modulus quantified as the slope of the plot linear region at 25-30% strain.

### Degradation Tests

Dry samples (n = 4) of either UV-cured AC network or native AC were incubated for 4 days (37°C, pH 7.5) in 1 mL of 50 mM [tris(hydroxymethyl)-methyl-2-aminoethane sulfonate] (TES) buffer containing 0.36 mM calcium chloride and supplemented with 5 CDU of collagenase type I from *Clostridium histolyticum* (125 CDU·mg^−1^). Following 4-day incubation, the samples were washed in distilled water, dehydrated via an ascending series of ethanol solutions and air dried. The relative mass (μ_*rel*_) of samples was determined according to Equation 5:

(5) $$\begin{array}{l} \mu_{rel}=\frac{m_{4}}{m_{d}}\times 100 \end{array}$$

where *m*_4_ and *m*_*d*_ are the masses of the dry partially-degraded and dry freshly-synthesized samples, respectively.

### UV-Curing Rheological Measurements

The UV-induced kinetics of network formation (*n* = 3) was measured by a modular compact rotational rheometer (MCR 302, Anton Paar, Austria) equipped with a UV curing module (Ominicure 1500, Excelitus Technologies). Functionalized atelocollagen products (0.8 wt.%) were dissolved in I2959-supplemented aqueous solutions (1 wt.% I2959), followed by time sweep measurement at a strain of 0.1% and frequency of 1 Hz. Values of storage (*G*′) and loss (*G*″) modulus were recorded during time sweep measurements under irradiation with UV light. The oscillatory shear was applied to a transparent glass parallel plate (Ø 25 mm) and the gap between the plates was 300 μm. UV light (365 nm, 8 mW·cm^−2^) was initiated after 5 s of shear oscillation at 21°C. The gelation time (τ) was determined by the temporal interval between the UV activation and complete gelation.

### Cell Culture Study

G292 cells were cultured in Dulbecco's modified Eagle's medium (DMEM), supplemented with 10% fetal bovine serum (FBS), 1% glutamine, and 2.5 mg·mL^−1^ penicillin–streptomycin, in a humidified incubator at 37°C and 5% CO_2_. Cells were passaged every 3 days with 0.25% trypsin/0.02% EDTA. UV-cured samples were individually synthesized on to a 24-well plate, extensively washed in distilled water and dehydrated in an ascending series of ethanol-distilled water [0, 20, 40, 60, 80, (3×) 100 vol.% EtOH] to remove any acidic or ethanol residues. Prior to cell seeding, hydrogels were disinfected in a 70 vol.% ethanol solution under UV light and washed in PBS (3×, 10 min) to remove any acidic or ethanol residues. G292 cells (8·10^3^ cells·mL^−1^) were seeded on top of the sample (following UV disinfection) and incubated at 37°C for up to 7 days. After incubation, samples (*n* = 6) were washed with PBS (×3) and transferred to a new 24-well plate before adding the dying agent of Calcein AM and Ethidium homodimer-1. The sample plate was then incubated for 20 minutes away from light. Live /dead stained hydrogels were placed onto a glass slide for fluorescence microscopy imaging (Leica DMI6000 B).Cells grown on tissue culture treated plastics were used as positive control (Nunc, UK). Other than live/dead staining, cell viability was assessed at selected time points using Alamar Blue assay (ThermoFisher Scientific, UK) according to the manufacturer's guidance. VP-SEM (Hitachi S-3400N VP) combined with Deben cool stage control (Model: LT3299) was also employed for high resolution imaging of cell attachment on hydrated samples after 7-day incubation under low pressure (60–70 Pa).

### Statistical Analysis

Data are presented as mean ± standard deviation (SD). Statistical analysis was carried out with the Student's *t*-test. A *p* value lower than 0.05 was considered to be significantly different.

## Results and Discussion

In the following, the synthesis and characterization of UV-cured hydrogel networks made of either 4-vinylbenzylated or methacrylated atelocollagen is presented. The effects of covalently-coupled monomer, degree of atelocollagen functionalization and UV-curing aqueous solution was investigated, aiming to draw controlled structure-property relationships. The synthesis of the covalent networks was linked to the degree of functionalization of respective photoactive AC solutions, whilst respective UV-induced gelation kinetics was assessed in either acidic or basic conditions. Resulting UV-cured networks were characterized with regards to rheological properties, gel content, swelling ratio, compression modulus, enzymatic degradability, and cellular tolerability.

The sample nomenclature is as follows: functionalized AC samples are coded as “XXXYY,” where “XXX” identifies the type of monomer introduced on to the AC backbone, i.e., either 4VBC or MA; and “YY” describes the monomer/Lys molar ratio used in the functionalization reaction. UV-cured samples are coded as “XXXYY(Z)^*^,” where “XXX” and “YY” have the same meaning as previously discussed; “Z” indicates the UV-curing aqueous solution used to dissolve the functionalized AC sample, i.e., either 10 mM HCl (H), 17.4 mM acetic acid (A) or 10 mM PBS (P); “^*^” identifies a UV-cured hydrogel sample.

### Synthesis of Functionalized AC Precursors

Following reaction with either 4VBC or MA, the degree of functionalization (*F*) consequent to the covalent coupling of photoactive adducts on to the AC backbone was determined. Since network formation was pursued via UV-induced free radical crosslinking mechanism, the introduction of photoactive adducts was expected to be directly related to the crosslink density and macroscopic properties of respective AC networks. The reaction of AC with selected monomers proceeds via lysine-initiated nucleophilic substitution (Figure S1) leading to the consumption of free amino groups and derivatization with either 4-vinylbenzyl or methacrylamide adducts. TNBS (Bubnis and Ofner, 1992) and ninhydrin (Starcher, 2001) assays are two colorimetric assays widely employed for the determination of amino groups in proteins. Both assays have recently been employed for the characterization of reacted gelatin (Billiet et al., 2013; Kishan et al., 2015) and acid-solubilized collagen (Ravichandran et al., 2016; Tronci et al., 2016a,b; Liang et al., 2018) products, proving to correlate well with ^1^H-NMR spectroscopy. Both TNBS and ninhydrin assays were therefore selected in this study to measure the molar content of free amino groups in both native and reacted pepsin-solubilized AC samples, so that *F* could be indirectly quantified.

An overall content of primary amino groups of 2.89·10^−4^ mol·g^−1^ was recorded on native atelocollagen via TNBS assay. Both colorimetric methods revealed a comparable and decreased molar content of amino groups in both MA- and 4VBC-reacted atelocollagen samples (Table 1), in line with previous reports (Jia et al., 2012; Tronci et al., 2016b); whilst monomer-related geminal protons could not be clearly detected in ^1^H-NMR spectra of sample MA0.3 and 4VBC25 due to the overlapping with AC species (Figure S2). The range of MA/Lys molar ratio selected during the functionalization reaction proved to directly impact on the degree of atelocollagen methacrylation (*F*_*MA*_ = 4 ± 1 – 88 ± 1 mol.%), whilst an insignificant variation in the molar content of covalently-coupled 4VBC adducts (*F*_4*VBC*_ = 18 ± 1 mol.%) was observed in respective products (data not shown). The different trend in degree of functionalization observed in MA- with respect to 4VBC-reacted samples is attributed to the decreased reactivity and solubility of 4VBC in aqueous environment, as reported in previous publications (Tronci et al., 2016a), which was partially mitigated in this study by the addition of PS-20 in the reacting mixture.

**Table 1:** TNBS assay quantification of the amino group molar content and degree of functionalization (*F*) in atelocollagen products (*n* = 2) following reaction with either 4VBC or MA at varied monomer/Lys molar ratios.

**Sample ID**	**Molar ratio / [Monomer][Lys]^**−1**^**	**Amine groups/mol·g^−1^ (×10^−4^)**		**F */mol.% ^**(a)**^***
		**TNBS**	**Ninhydrin**
4VBC25	25	2.38 ± 0.02	2.38 ± 0.01	18 ± 1
MA0.1	0.1	2.77 ± 0.03	n.a.	4 ± 1
MA0.3	0.3	2.34 ± 0.06	2.40 ± 0.13	19 ± 2
MA0.5	0.5	2.07 ± 0.14	n.a.	28 ± 5
MA1	1	1.63 ± 0.11	n.a.	44 ± 4
MA25	25	0.36 ± 0.01	0.44 ± 0.04	88 ± 1

*The quantification of amino group molar content was confirmed via Ninydrin assay (n = 2). n.a., not applicable*.

(a) *F was calculated considering an overall molar content of amino groups of 2.89 × 10^–4^ mol·g^–1^ in native AC, as revealed by TNBS assay*.

Despite the different reactivity of MA with respect to 4VBC, the range of *F* observed in resulting MA-functionalized AC is in line with the results reported in previous publications (Brinkman et al., 2003; Gaudet and Shreiber, 2012; Ravichandran et al., 2016) with collagen-based materials.

Billiet et al. analyzed the covalent coupling of (meth)acrylic anhydride to bovine type B gelatin and achieved up to ca. 80 mol.% methacrylation (Billiet et al., 2013). Ravichandran et al. reported a degree of porcine collagen methacrylation of 57–87 mol% (Ravichandran et al., 2016), and similar results (*F* = 39–86 mol.%) were obtained by Chaikof et al. with acid-soluble rat tail collagen (Brinkman et al., 2003). Compared to the latter case, the results presented in this work with pepsin-soluble collagen suggest that the removal of telopeptides does not significantly impact on the molar content of free amino groups and functionalization capability of resulting atelocollagen product, in line with a previous report (Tronci et al., 2016b).

In order to systematically investigate how above-mentioned differences at the molecular scale of AC precursors were reflected on the macroscale of resulting UV-cured networks, it was therefore of interest to systematically vary either the type of covalently-coupled monomer (whilst keeping respective degree of AC functionalization constant) or the degree of functionalization (with the same type of covalently-coupled monomer). Accordingly, samples MA0.3 (*F*_*MA*_ = 19 ± 2 mol.%), 4VBC25 (*F*_4*VBC*_ = 18 ± 1 mol.%), and MA25 (*F*_*MA*_ = 88 ± 1 mol.%) were selected for further investigations, and dissolved in either hydrochloric acid, acetic acid or PBS, as commonly-used solvent for collagen, prior to UV curing.

### UV-Induced Network Formation and Time-Sweep Photorheometry

**Figure 1:**
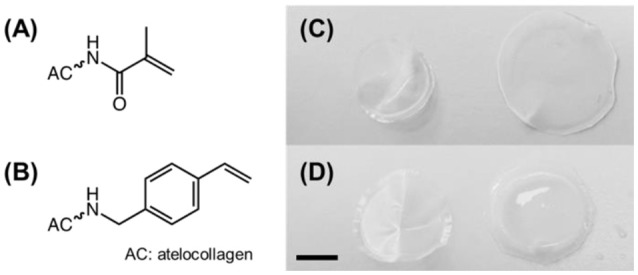
**(A,B)** Structural formula of methacrylamide **(A)** and 4-vinylbenzyl **(B)** residues following covalent coupling to atelocollagen. **(C,D)** Digital macrographs of UV-cured networks MA0.3(H)* **(C)** and 4VBC25(H)* **(D)** in both dry (left) and water-equilibrated (right) states. Scale bar: 5 mm.

AC samples functionalized with methacrylamide residues (Figure 1A) formed a clear solution in both acidic and PBS solutions, whilst sample 4VBC25 (Figure 1B) could only be dissolved in 10 mM HCl (pH 2.1) and 17.4 mM AcOH (pH 3.4). The insolubility of the 4VBC-Functionalized AC product in PBS agrees with previous reports (Tronci et al., 2015a) and may be attributed to secondary interactions developed between aromatic 4-vinylbenzyl residues following atelocollagen functionalization and sample drying. Aromatic rings can mediate π-π stacking interactions as well as act as hydrogen bond acceptor in aqueous environment (Levitt and Perutz, 1988). Resulting π-π stacking interactions developed between 4VBC-functionalized AC molecules are likely to be broken down at decreased rather than basic pH, supporting the observed complete dissolution of sample 4VBC25 in both HCl and AcOH solutions.

**Figure 2:**
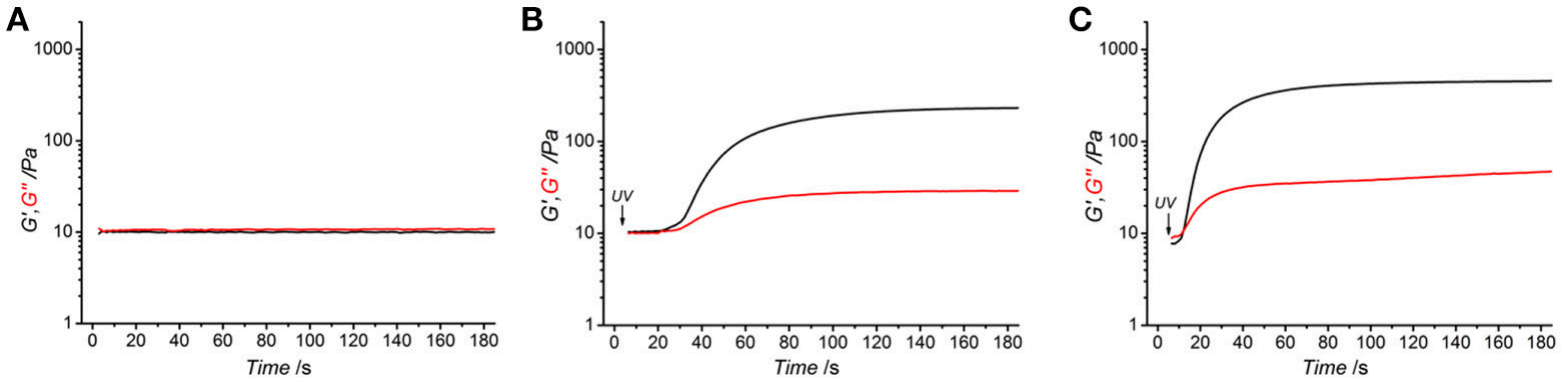
Rheograms of functionalized atelocollagen solution (0.8 wt.%) of sample 4VBC25 in 17.4 mM acetic acid supplemented with either 1 wt.% I2959 **(A,C)** or 0.5 wt.% I2959 **(B)**. **(A)** Storage (*G*′, black) and loss (*G*″, red) modulus recorded in the absence of UV light. **(B,C)**: UV-induced gelation kinetics of functionalized atelocollagen solutions. UV light was activated 5 s following shear oscillation.

All photoactive solutions successfully yielded hydrogel networks when treated with UV light, irrespective of the type of monomer introduced on to AC (Figures 1C,D). To confirm the formation of a UV-cured hydrogel network and to explore the effect of methacrylamide and 4-vinlybenzyl residues on gelation kinetics, time sweep photorheometry was carried out prior to and during UV irradiation of AC photoactive solutions, and changes in viscosity (η) as well as storage (*G*′) and loss (*G*″) modulus recorded. Prompt increase of *G*′ and *G*″ was observed following activation of the atelocollagen mixtures with UV light, in contrast to the case of the same sample being tested in the absence of UV light (Figure 2). Comparable values of *G*′ and *G*″ were also measured in the latter case, providing indirect evidence of the absence of a crosslinked network at the molecular level (Table 2). Both the gelation, i.e., the time required for the storage and loss moduli to equate, and plateau in storage modulus were reached within 12 s and 180 s, respectively (Table 2). The photoinitiator concentration (1 wt.%) selected to prepare gel-forming AC solutions proved key to reduce gelation time and to generate hydrogels with increased elastic modulus with respect to gel-forming AC solutions supplemented with decreased (0.5 wt.%) photoinitiator concentration (Figures 2B,C).These results, together with the fact that the final values of *G*′ were significantly higher compared to the values of *G*″ (Table 2), successfully indicate complete conversion of the photoactive solution into a UV-cured covalently-crosslinked network (McCall and Anseth, 2012). The obtained gelation kinetics curves are comparable to the ones described by thiol-ene collagen- poly(ethylene glycol) mixtures (Holmes et al., 2017), whereby gel points were recorded within ~7 s of UV activation. This observation is interesting given that the orthogonality and oxygen-insensitivity of the UV-induced thiol-ene, with respect to the free-radical, crosslinking reaction, should be corelated with decreased gelation times (Van Hoorick et al., 2018).

**Table 2:** Viscosity (η) and gelation time (τ) of hydrogel-forming solution as well as storage (*G*′) and loss (*G*″) moduli of UV-cured hydrogel networks following complete gelation.

**Sample ID**	**η /mPa·s ^**(1)**^**	**G′_max_ /Pa**	**G″_max_ /Pa**	**τ /s**
AC^(2)^	1777 ± 20	16 ± 1	13 ± 1	22 ± 5
4VBC25(H)^*^	900 ± 70^(a)^	73 ± 22^(b)^	20 ± 3^(b)^	10 ± 0^(a)^^(d)^^(c)^
4VBC25(A)^*^	1820 ± 80	390 ± 99	42 ± 7	8 ± 0 ^(a)^
MA0.3(H)^*^	390 ± 20^(a)^^(c)^	17 ± 3^(a)^	8 ± 5^(a)^	12 ± 1^(d)^^(c)^
MA0.3(A)^*^	240 ± 20^(a)^	58 ± 5^(a)^	17 ± 3^(d)^	11 ± 1
MA0.3(P)^*^	240 ± 20^(c)^	85 ± 1^(a)^	24 ± 2^(a)^^(d)^	11 ± 1
MA25(H)^*^	1200 ± 130	12967 ± 265^(a)^	42 ± 4^(a)^^(b)^	6 ±1^(c)^
MA25(A)^*^	920 ± 100^(d)^	618 ± 87^(a)^^(b)^	8 ± 1^(a)^^(c)^	n.a.
MA25(P)^*^	1400 ± 190	18897 ± 4793^(b)^	146 ± 366^(b)^^(c)^	6 ± 0

*Data are presented as mean ± SD (n = 3)*.

(1) *Viscosity of hydrogel-forming AC solutions*.

(2) *Atelocollagen (0.8 wt.%) solution control prepared in 17.4 mM acetic acid supplemented with 1 wt.% I2959*.

(a)&(c) *p < 0.001*;

(b) *p < 0.01*;

(d) *p < 0.05*.

**Figure 3:**
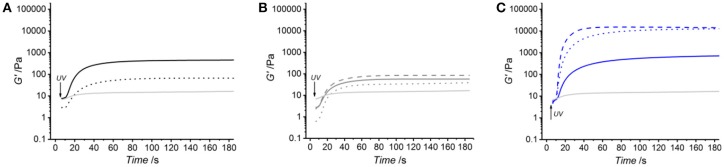
Kinetics of network formation studied via UV-equipped rheometry on samples 4VBC25 **(A)**, MA0.3 **(B)** and MA25 **(C)**, when dissolved (0.8 wt.%) in either 10 mM hydrochloric acid, 17.4 mM acetic acid or 10 mM PBS solution (each supplemented with 1 wt.% I2959). UV light was activated following 5 s shear oscillation. **(A)**: (···): 4VBC25(H)^*^; (—): 4VBC25(A)^*^. **(B)**: (···): MA0.3(H)^*^; (—): MA0.3(A)^*^; (– – –): MA0.3(P)^*^. **(C)**: (···): MA25(H)^*^; (—): MA25(A)^*^; (– – –): MA25(P)^*^. A solution of atelocollagen (0.8 wt.%) in 17.4 mM acetic acid (supplemented with 1 wt.% I2959) was also tested and respective *G*′ profile reported in each plot (light gray curve).

Figure 3 and Table 2 describe the gelation kinetic profiles of samples 4VBC25, MA0.3, and MA25 when dissolved in either 10 mM HCl, 17.4 mM AcOH or 10 mM PBS solutions supplemented with 1 wt.% I2959, whilst an atelocollagen control sample was also tested following solubilization in a 17.4 mM acetic acid solution supplemented with 1 wt.% I2959. Overall, sample MA25 was found to generate networks with the highest loss and storage modulus (*G*′ = 618 ± 87 – 18897 ± 4793 Pa) and fastest gelation kinetics (τ ~ 6 s), followed by samples 4VBC25 (*G*′ = 73 ± 22 – 390 ± 99 Pa) and MA0.3 (*G*′ = 17 ± 3 – 85 ± 1 Pa). In contrast, only a marginal increase of *G*′ was observed following UV activation of the control atelocollagen solution, likely due to the much lower radical-induced crosslinking of amino acidic residues (Heo et al., 2016) with respect to the complete UV-induced network formation achieved with functionalized atelocollagen precursors. This result therefore demonstrates the importance of selected functionalization routes in ensuring the synthesis of full covalent networks and provides additional evidence of the covalent coupling of selected monomers to atelocollagen. Other than the variation in elastic modulus, the viscosity of respective network-forming AC solutions was also recorded, whereby the sample MA25 proved to generate highly viscous solutions in all solutions investigated (η = 920 ± 100 – 1400 ± 190 mPa·s). The variations observed in loss and storage modulus and gelation kinetics support the trends in degree of functionalization recorded at the molecular scale of the atelocollagen precursors, whereby sample MA25 exhibited the highest molar content of photoactive residues with respect to the other two samples. Given that the covalently-coupled photoactive adducts mediate the UV-induced free-radical crosslinking reaction, the increased storage and loss moduli as well as decreased gelation time measured in these samples therefore provide indirect confirmation that changes in the degree of AC functionalization directly impact on the crosslink density, gelation kinetics and elastic properties of resulting UV-cured networks. The increased solution viscosity recorded with MA25 also correlates with respective degree of methacrylation, due to the increased molar content and molecular weight of derivatized lysine terminations.

Together with the effect of varied degrees of functionalization, equimolar functionalization of AC with distinct photoactive adducts, i.e., either methacrylamide or 4-vinylbenzyl residues, was also found to play a detectable effect on the macroscopic properties of resulting UV-cured hydrogels (Table 2). Both networks 4VBC25(H)^*^ and 4VBC25(A)^*^ displayed significantly increased storage and loss moduli with respect to the methacrylated equivalents (prepared in the same solvents). Also in this case, the trend in mechanical properties was supported by direct variations in respective network-forming solutions, whereby significantly-increased solution viscosity and decreased gelation times were measured with sample 4VBC25 (η = 900 ± 70 – 1820 ± 80 mPa·s; τ = 8 ± 0 – 10 ± 0 s) compared to sample MA0.3 (η = 240 ± 20 – 390 ± 20 mPa·s; τ = 11 ± 1 – 12 ± 1 s).

The significant difference in storage and loss modulus measured in UV-cured aromatized atelocollagen networks with respect to equivalent methacrylated variants gives evidence of the major role played by the monomer covalently-coupled to, and resulting photocrosslinked segment between, AC molecules. Aromatic interactions have been exploited for the formation of physically-crosslinked, self-assembled peptides (Birchall et al., 2011), and for the controlled delivery of hydrophobic drugs (Li et al., 2010). The above-mentioned increased viscosity, storage modulus and loss modulus measured on 4VBC-functionalized atelocollagen solutions and corresponding UV-cured networks can mostly be attributed to π-π stacking interaction capability and increased molecular rigidity of aromatic 4VBC moieties and consequent UV-cured aromatized segment, respectively. Other than that, the decreased gelation time recorded in solutions of sample 4VBC25 with respect to solutions of sample MA0.3 seems to correlate with the increased segment length of the 4-vinylbenzyl, with respect to methacrylamide, moiety (Figure 1A), so that crosslinking-hindering steric effects are less likely in the former compared to the latter system. Rather than the typical variation of the molar content of photoactive groups, these results therefore demonstrate the possibility of adjusting the mechanical properties of atelocollagen hydrogels by simply varying the type of monomer covalently-coupled to the AC backbone.

Other than the degree and type of functionalization, the effect of the UV-curing aqueous solution was also explored as an additional parameter to control material behavior (Figure 2, Table 2). Hydrochloric acid solutions (10 mM, pH = 2.1) of AC precursors functionalized with equimolar content of either 4VBC or MA adducts yielded crosslinked hydrogels with the lowest storage (G′_4*VBC*25_ = 73 ± 22 Pa; G′_*MA*0.3_ = 17 ± 3 Pa) and loss (G″_4*VBC*25_ = 20 ± 3 Pa; G″_*MA*0.3_ = 8 ± 5 Pa) moduli, compared to hydrogels prepared from atelocollagen solutions in acetic acid (17.4 mM, pH 3.4) and PBS (10 mM, pH 7.5). Interestingly, similar trends were observed in the case of 4VBC-functionalized AC solutions, whereby significantly-increased viscosities were measured when the photoactive precursor was dissolved in hydrochloric acid (η = 900 ± 70 mPa·s) compared to acetic acid (η = 1820 ± 80 mPa·s) solutions (Table 2), whilst an opposite trend was observed in acidic solutions prepared with samples MA0.3 and MA25.

The solution-induced effect observed in AC products functionalized with equivalent molar content of methacrylamide and 4-vinylbenzyl residues is in agreement with the results reported by Ratanavaraporn et al. (2008), whereby increased solution viscosity and hydrogel compressive modulus were measured when native type I collagen was dissolved in aqueous solutions of decreased acidity. It is well known that the molecular organization of native collagen molecules can be altered depending on environmental factors, such as pH, ionic strength and salt concentration, given their effect on the ionization of amino acidic terminations and consequent secondary interactions (Salchert et al., 2004; Yunoki and Matsuda, 2008; Grant et al., 2009; Neel et al., 2013). The above-mentioned variations in network elastic properties suggest that a similar effect can still be observed in the case of functionalized atelocollagen with decreased *F*. Whilst fibrillogenesis was not expected during network formation (*T* < 37°C), the electrostatic repulsion of native atelocollagen molecules is expected to be increased in solutions of increased acidity (Ratanavaraporn et al., 2008; Li et al., 2009). Obviously, the degree and type of monomer adds further complexity to the solution-induced variability of protein organization, given that the atelocollagen functionalization is achieved by the consumption of ionisable lysine terminations and that selected covalently-coupled adducts can mediate further secondary interactions, i.e., π-π stacking interactions and hydrogen bonds. The opposite pH-induced variation in solution viscosity measured with samples 4VBC25, on the one hand, and MA0.3 and MA25, on the other hand, reflects these considerations, given that aromatic interactions between 4-vinylbenzyl moieties are likely to be decreased at increased pH, whilst an opposite trend is expected with regards to the hydrogen bonding capability of methacrylamide residues. Whilst a solution pH-induced effect was clearly observed in hydrochloric and acetic acid solutions, the presence of salts in the PBS-based UV-curing system was likely to alter the capability of covalently-coupled moieties to mediate secondary interactions, explaining why no direct relationships between solution pH, on the one hand, and solution viscosity and hydrogel properties, on the other hand, could be observed.

### Swelling, Gel Content and Compression Properties

Following the characterization of functionalized AC precursors and gelation kinetics, the gel content (*G*), swelling ratio (*SR*) and compressive properties were quantified to further elucidate the molecular architecture and assess the structure-property relationships of obtained UV-cured atelocollagen networks.

**Figure 4:**
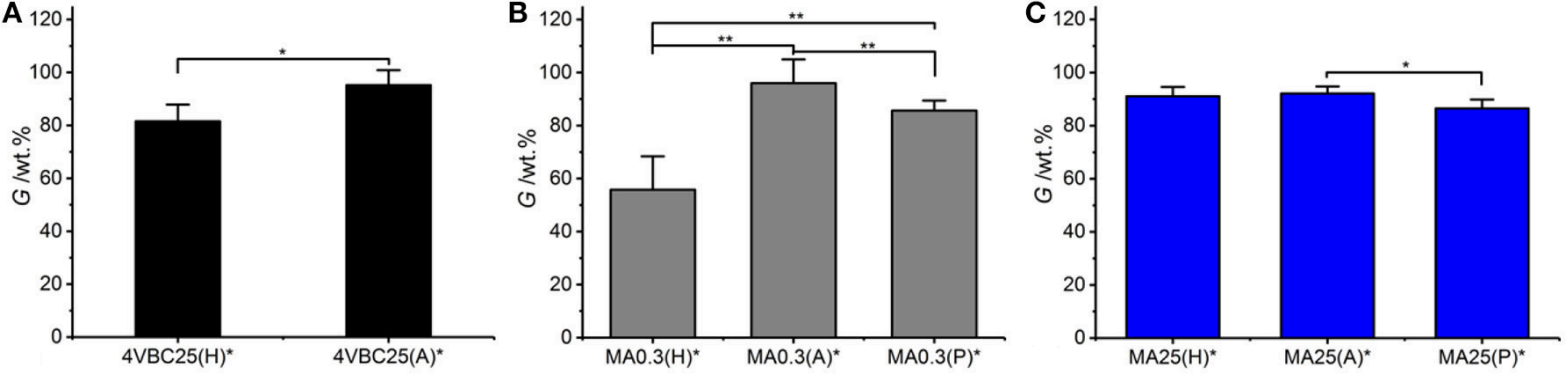
Gel content (*G*) of atelocollagen networks 4VBC25* **(A)**, MA0.3* **(B)**, and MA25* **(C)** prepared in either 10 mM hydrochloric acid, 17.4 mM acetic acid, or 10 mM PBS solution. **p < 0.01 and *p < 0.05.

All UV-cured networks displayed an averaged gel content of at least 80 wt.%, with the exception of sample MA0.3(H)^*^ (*G* = 56 ± 13 wt.%) (Figure 4). Samples MA25^*^ displayed the smallest variation in gel content when prepared in either HCl, AcOH, or PBS solutions, whilst the effect of the UV-curing solution was more visible in samples of MA0.3^*^ and 4VBC25^*^. The high, and small solution-induced variation of, gel content measured in samples MA25^*^ confirms that precursors with increased degree of functionalization generate highly crosslinked network, regardless of the type of UV-curing solution and solution-induced secondary interactions, as indicated previously (Tables 1, 2).

In comparison to sample MA25^*^, networks 4VBC25(H)^*^, and MA0.3(H)^*^ prepared in HCl solutions revealed the lowest gel content within the same sample group, whilst increased *G* values were measured in networks prepared in either AcOH, i.e., 4VBC25(A)^*^ and MA0.3(A)^*^, or PBS, i.e., MA0.3(P)^*^. Given the comparable degree of functionalization in the network precursors (Table 1), these solution-induced variations in gel content seem to correlate with the above-mentioned results in UV-curing solution viscosity (Table 2), whereby an increase of solution pH from 2.1 (in 10 mM HCl) to 3.4 (in 17.4 mM AcOH) proved to generate hydrogels 4VBC25^*^ with increased storage modulus (Table 2). An increase in solution viscosity is likely to be associated with an increased vicinity between atelocollagen molecules and respective crosslink-forming photoactive residues, so that increased chances of free-radical crosslinking reaction and minimized steric hindrance effects can be expected. Similar effects were also reported with collagen hydrogels prepared from solutions with decreased viscosity, whereby decreased mechanical properties and increased pore size was observed (Ratanavaraporn et al., 2008).

**Figure 5:**
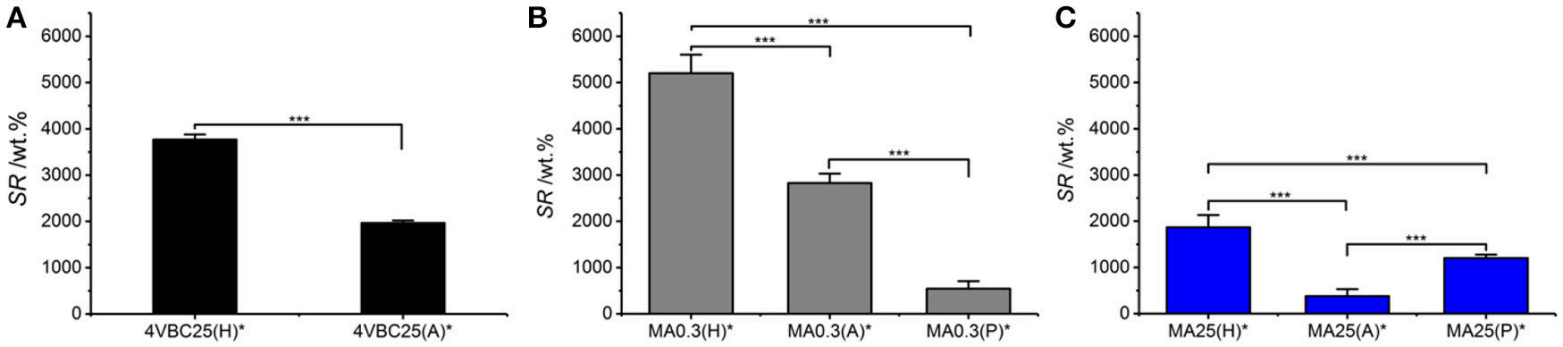
Swelling ratio (SR) of atelocollagen networks 4VBC25* **(A)**, MA0.3* **(B)**, and MA25* **(C)** prepared in either 10 mM hydrochloric acid, 17.4 mM acetic acid, or 10 mM PBS solutions. ***p < 0.001.

Previously-discussed results in gel content proved to support the trends in swelling ratio (Figure 5), whereby UV-cured networks described an inverse relationship between *G* and *SR*. For example, samples 4VBC25(H)^*^ (*SR* = 3769 ± 111 wt.%) and MA0.3(H)^*^ (*SR* = 5202 ± 401 wt.%) displayed the highest swelling ratio, confirming the higher water uptake capability of these materials with respect to sample variants prepared in AcOH solutions, reflecting above-discussed gel content trends.

Among the methacrylated groups, networks MA25^*^ displayed the lowest value of *SR* (Table 1, Figure 5). Samples MA25^*^ UV-cured in both AcOH and HCl solutions proved to display significantly decreased *SR* with respect to the ones of respective sample MA0.3^*^, in line with previous gel content data. On the other hand, an opposite trend was observed in networks synthesized in PBS, whereby sample MA25(P)^*^ displayed higher *SR* with respect to sample MA0.3(P)^*^, despite the comparable gel content between the two samples (Figure 4) and the significantly higher degree of functionalization displayed by the former compared to the latter network precursor. Given that the swelling ratio was determined in PBS, the reason for this finding is likely explained by the fact that the increased molar content of methacrylamide moieties covalently-coupled to sample MA25 results in additional secondary interactions of the resulting network with PBS species, in line with the viscosity trends measured with the hydrogel-forming solutions (Table 2).

Overall, the water uptake capability of these atelocollagen networks was found to be comparable to the one of purely synthetic UV-cured PEG-based hydrogels (DiRamio et al., 2005) and may be exploited for the design of multifunctional wound healing devices with enhanced exudate regulation capability (Bulman et al., 2015; Tronci et al., 2016b). The possibility to adjust the swelling properties of this atelocollagen system by simply varying the UV-curing solution rather than by the synthesis of new photoactive precursors is appealing aiming to expand material applicability yet minimizing additional time-consuming reactions.

**Figure 6:**
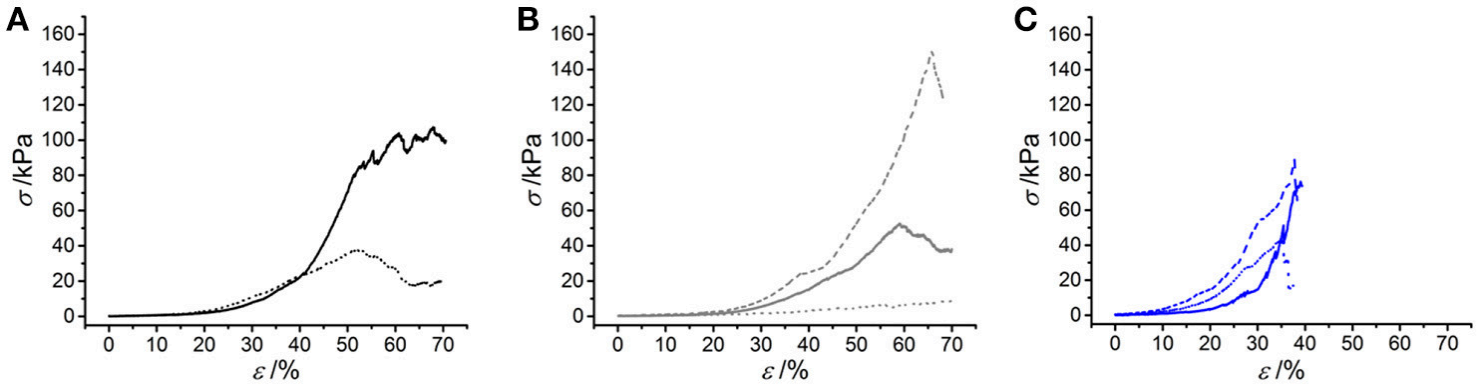
Typical stress-compression curves of hydrogels 4VBC25* **(A)**, MA0.3* **(B)** and MA25* **(C)** prepared in either 10 mM hydrochloric acid (pH 2.1), 17.4 mM acetic acid (pH 3.4) or 10 mM PBS (pH 7.5) solution. **(A)**: (···): 4VBC25(H)*; (—): 4VBC25(A)*. **(B)** (···): MA0.3(H)*; (—): MA0.3(A)*; (– – –): MA0.3(P)*. **(C)** (···): MA25(H)*; (—): MA25(A)*; (– – –): MA25(P)*.

**Figure 7:**
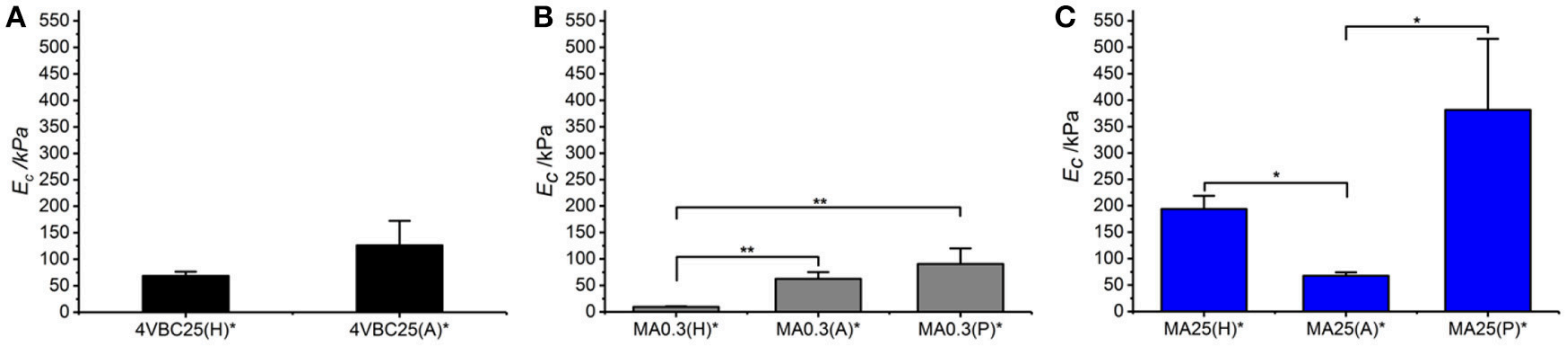
Compressive modulus (*E*_*c*_) of atelocollagen hydrogels 4VBC **(A)**, MA0.3 **(B)**, and MA25 **(C)**, prepared in varied solvents. Data are presented as mean ± SD (*n* = 3). **p < 0.01, *p < 0.05.

Following characterization of the swelling behavior, the compressive properties of resulting UV-cured hydrogels were investigated. Typical stress-compression curves are reported in Figure 6, depending on the network architecture and type of UV-curing solvent employed. All samples revealed a *J*-shaped curve during compression, similarly to the case of previously-reported collagen hydrogels (Yunoki and Matsuda, 2008). Networks MA25^*^ proved to display lower values of compression at break compared to both networks 4VBC25^*^ and MA0.3^*^ (Figure 7), in line with the increased degree of functionalization (*F* = 88 ± 1 mol.%) of the former network precursors, yielding highly crosslinked networks (*G* = 86 ± 3 – 92 ± 3 wt.%). When comparing systems with comparable degrees of functionalization, samples 4VBC25^*^ displayed a detectable increase in compression modulus (*E*_*c*_ = 69 ± 8 – 126 ± 46 kPa) when compared to samples MA0.3^*^ (*E*_*c*_ = 9 ± 1 – 62 ± 13 kPa), in all environmental conditions investigated. Among the different aqueous media, UV-curing in HCl solutions was confirmed to yield networks with the lowest compression modulus (Figure 7), supporting previous rheological (Table 2), and gel content (Figure 4) measurements.

Remarkably, sample 4VBC25(H)^*^ proved to display an averaged compressive modulus of about 70 kPa, despite an averaged swelling ratio of more than 3700 wt.%. In comparison, an equivalent methacrylated network exhibited a much lower averaged compressive modulus of less than 9 kPa, supporting the low gel content (*G* = 56 ± 13 wt.%) and high swelling ratio (*SR* = 5202 ± 401 wt.%). The uniquely high compressive modulus *and* swelling ratio of networks 4VBC25^*^ provides additional evidence of the key role played by the molecular stiffness of the 4VBC-based crosslinking segment, in comparison to equivalent methacrylated atelocollagen networks obtained from precursors with comparable degree of functionalization.

When comparing systems with varied degree of functionalization, samples MA25^*^ proved to display significantly increased compressive modulus with respect to samples MA0.3^*^ (and 4VBC25^*^), confirming the previous trends in storage modulus (Table 2). The effect of the solvent in samples MA25^*^ proved to be less obvious, as indicated by the variation of gel content and swelling ratio. On the one hand, the significant increase in molar content of methacrylamide residues is likely to generate UV-cured networks with increased crosslink density. On the other hand, significant atelocollagen methacrylation is expected to alter the protein capability to mediate secondary interactions, potentially resulting in altered structure-function relationships. Therefore, rather than aiming at increased degree of methacrylation, these results suggest that the introduction of a limited amount of stiff 4-vinylbenzyl moieties is preferable in order to achieve defined mechanically-competent atelocollagen hydrogels.

### Enzymatic Degradability

**Figure 8:**
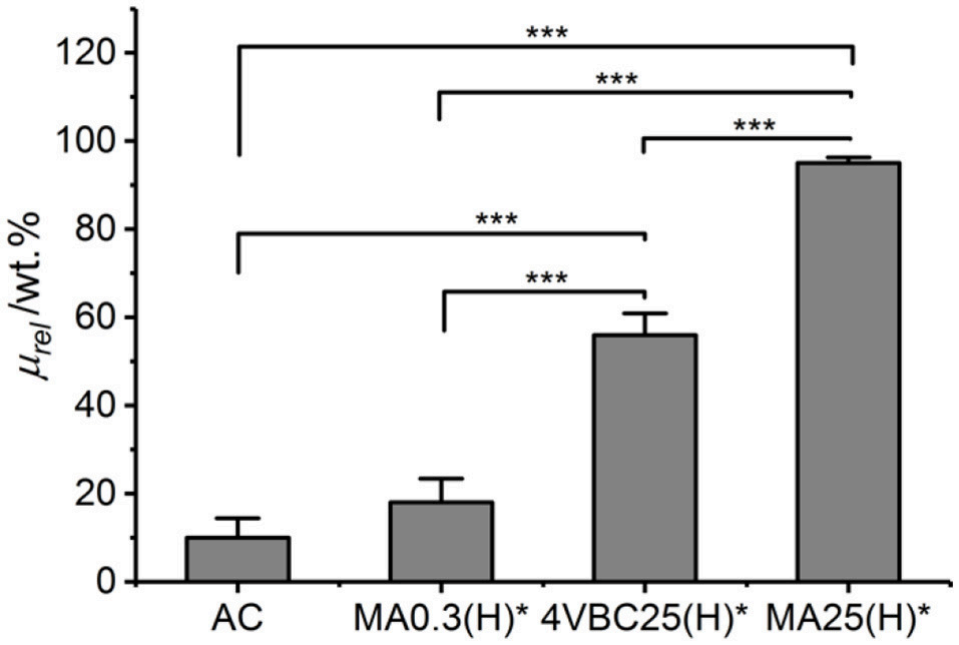
Relative mass (μ_*rel*_) recorded following 4-day hydrogel incubation in a collagenase-containing aqueous solution (5 CDU, 37°C, pH 7.5). AC is the control sample of native atelocollagen. Data are expressed as mean ± SD (*n* = 4). ***p < 0.001.

A degradation study was carried out in enzymatic conditions in order to investigate how variations in network architecture were linked to the temporal stability of the hydrogel in near-physiologic conditions. Atelocollagen networks UV-cured in HCl solutions were selected for this investigation, due to the significant differences observed between samples 4VBC25(H)^*^ and MA0.3(H)^*^. Figure 8 describes the relative mass results recorded following 4-day network incubation in an aqueous medium containing collagenase. As expected, samples of native atelocollagen revealed the highest enzymatic degradation with less than 10 wt.% remaining mass, followed by networks MA0.3H^*^ (μ_*rel*_ = 18 ± 5 wt.%) and 4VBC25H^*^ (μ_*rel*_ = 56 ± 5 wt.%), whilst the highest proteolytic stability was displayed by sample MA25H^*^ (μ_*rel*_ = 95 ± 1 wt.%).

Chemical crosslinking of collagen has been described as a promising strategy to increase the enzymatic stability of collagen, since the covalent crosslinks between protein molecules can be insensitive to collagenases and affect the availability of tripe helical segments recognizable by enzymes (Olde Damink et al., 1995a; Haugh et al., 2011; Van Vlierberghe et al., 2011; Lee et al., 2013; Duan et al., 2014; Tronci et al., 2016b; Calciolari et al., 2018). Therefore, aforementioned degradation data successfully support the effect played by the network architecture on the enzymatic stability of the hydrogel, both in terms of type of covalently-coupled monomer as well as consequent degree of atelocollagen functionalization. Samples MA25(H)^*^ displayed the highest enzymatic stability given the high degree of functionalization and gel content measured in network precursor and resulting crosslinked network, respectively. Other than that, controlled enzymatic degradation can still be achieved by simply varying the type of monomer introduced, i.e., 4-vinylbenzyl with respect to methacrylamide residue, whilst keeping the degree of functionalization constant, in resulting photoactive precursors. The introduction of 4VBC moieties is likely to mediate secondary interactions with the exposed zinc site of active collagenases (Tronci et al., 2016b), so that consequent proteolytic inactivation explains the prolonged stability of sample 4VBC(H)^*^ with respect to the equivalent variant MA0.3(H)^*^.

Similarly to the case of linear biopolymers (Lee et al., 2013), the manipulation of the atelocollagen network architecture is therefore proven to generate customizable hydrogels, whose degradation profiles can be combined with specific mechanical and swelling properties, depending on specific environmental (e.g., pH) and molecular parameters selected during UV-curing.

### Cytotoxicity Evaluation

**Figure 9:**
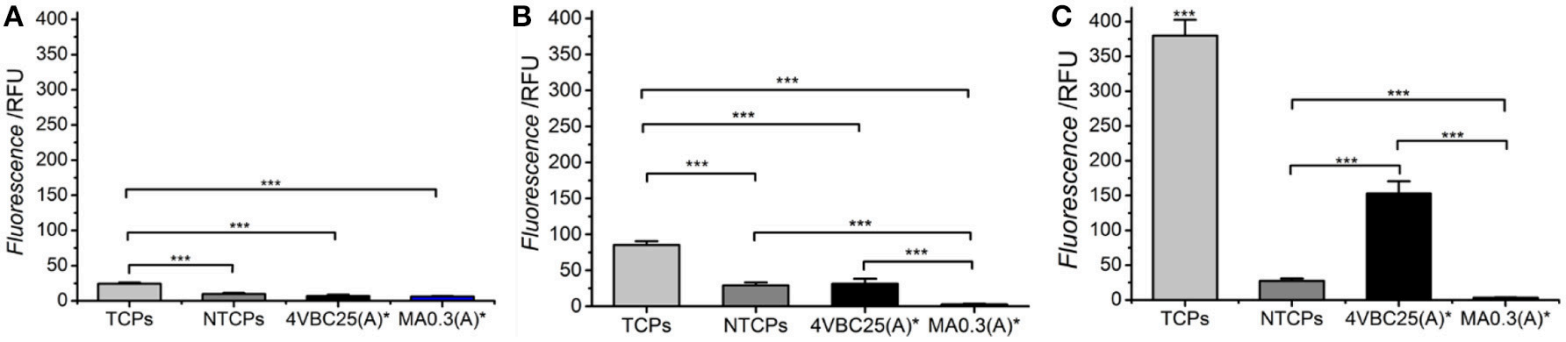
Alamar blue assay on G292 cells cultured on freshly-synthesized hydrogels 4VBC25(A)* and MA0.3(A)* at day 1 **(A)**, 4 **(B)**, and 7 **(C)**. Cells seeded on non-tissue culture treated plates (NTCPs) and tissue culture treated plates (TCPs) were used as either negative or positive control, respectively. ***p < 0.001 (*n* = 6).

Following evaluation of the enzymatic degradability, the cellular tolerability of freshly-synthesized networks 4VBC25(A)^*^ and MA0.3(A)^*^ was exemplarily evaluated by culturing osteosarcoma G292 cells in direct contact with the hydrated material. Cellular metabolism was measured by Alamar blue assay at day 1, 4, and 7 (Figure 9).

Over the period of 7 days, G292 cells seeded on TCPs displayed significantly increased proliferation at all time points investigated. Between the group of 4VBC25(A)^*^ and MA0.3(A)^*^, no significant difference was found at day 1; however, the proliferation rate of cells cultured on hydrogel 4VBC25(A)^*^ was much higher than the one of cells cultured on hydrogel MA0.3(A)^*^ at both day 4 and day 7. The dramatic turnover of cells on sample MA0.3(A)^*^ was mainly due to the shrinkage of the hydrogel microstructure observed already following 1-day cell culture, in line with the fast enzymatic sample degradability measured following 4-day incubation in collagenase medium (Figure 8). Unsurprisingly, TCPs showed the highest rate of proliferation compared to 4VBC25(A)^*^; this observation is mainly due to the fact that the porous 3D structure of the 4VBC25(A)^*^ hydrogel network induced low cell seeding efficiency and cellular penetration in comparison to 2D surfaces (Vunjak et al., 1998).

**Figure 10:**
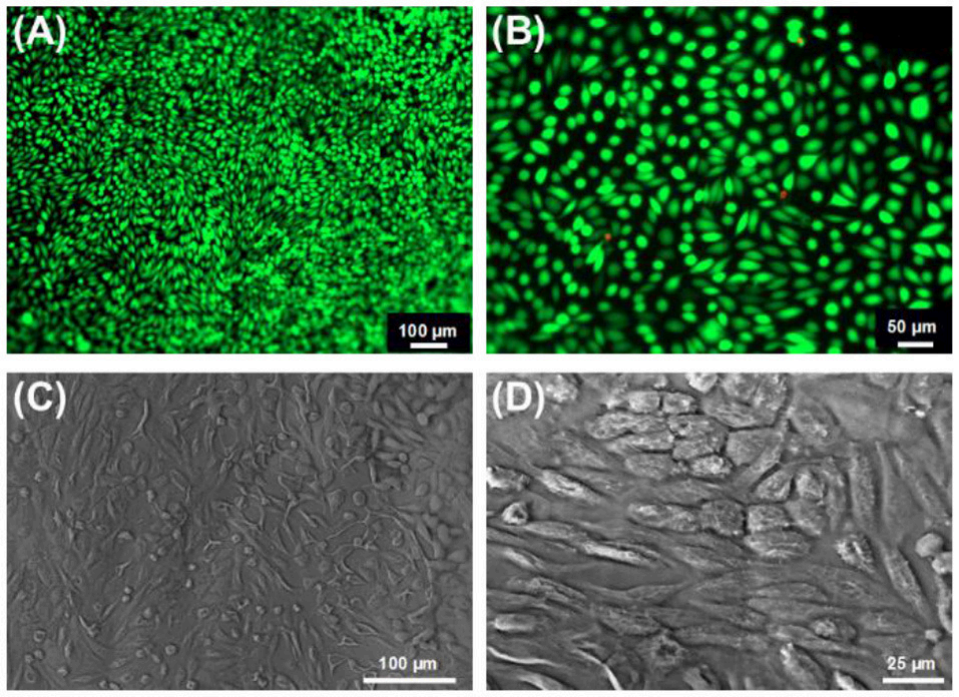
**(A,B)** Live/ dead staining of G292 cells following 7-day culture on hydrogels 4VBC25(A)*. **(C,D)**: Cell attachment was confirmed by cool stage SEM on the same sample at the same time point of cell culture.

We also confirmed the cytotoxicity of the hydrogels via Live/Dead staining of G292 cells following 7-day culture (Figures 10A,B). Almost no dead cell was found on hydrogel 4VBC(25)A^*^, indicating cellular tolerability in agreement with previously-discussed metabolic activity data. Besides Live/Dead staining, cool-stage SEM was also carried out on 7-day cultured hydrogel 4VBC25(A)^*^, as reported in Figures 10C,D, whereby full cellular attachment and complete surface cell coverage were observed, supporting previous findings. When the same SEM investigations were carried out on 7-day cultured hydrogel MA0.3(A)^*^, no cell attachment was observed (Figure S3), in line with Alamar blue results (Figure 9) and the fast enzymatic sample degradability (Figure 8).

Overall, these results prove that selected functionalization and network formation strategies did not affect atelocollagen biocompatibility, whilst proved successful in enabling systematic customization of the pristine building block.

## Conclusion

The introduction of either 4-vinylbenzyl or methacrylamide adducts onto type I atelocollagen proved successful to enable the synthesis of customizable UV-cured hydrogel networks, depending on the type of monomer, degree of atelocollagen functionalization, and UV-curing solution. Reaction with MA led to highly tunable degree of atelocollagen functionalization (*F*_*MA*_: 4 ± 1 – 88 ± 1 mol.%), in contrast to the reaction carried out with 4VBC (*F*_4*VBC*_: 18 ± 1 mol.%), although rapid gelation (τ = 6–12 s) was still achieved with both precursors as confirmed by photorheometry. Introduction of 4-vinylbenzyl groups proved to yield atelocollagen networks with significantly increased compression modulus (*E*_*c*_ = 69 ± 8 – 126 ± 46 kPa), storage modulus (*G*′ = 73 ± 22 – 390 ± 99 Pa) and 4-day enzymatic stability (μ_*rel*_ = 56 ± 5 wt.%), with respect to methacrylated equivalents, due to the increased molecular stiffness of, and secondary interactions mediated by, the aromatized UV-cured crosslinking segments. Comparable variations in material properties were also observed when UV-curing atelocollagen precursors functionalized with varied content of methacrylamide functions (*F* = 19 ± 2 – 88 ± 1 mol.%), supporting the direct relationships between the degree of functionalization of network precursors and resulting network crosslink density. The solution pH proved to affect the viscosity of respective atelocollagen solutions, whereby the monomer capability to mediate secondary interactions was found to play a role. UV-curing solutions with decreased acidity proved to generate networks with increased compression and storage modulus, as well as decreased swelling ratio, whilst no toxic cellular response was observed. These findings demonstrate the monomer-induced customizability of presented UV-cured atelocollagen hydrogel networks, whereby structure-property relationships can be controlled to enable applicability in personalized medicine and to fulfill the requirements of complex and unmet clinical needs.

## Author Contributions

HL and GT conceived and designed the experiments; HL performed the experiments. DW and SR assisted in the preparation of the manuscript. GT and HL wrote the paper. All authors analyzed the data.

### Conflict of Interest Statement

The authors declare that the research was conducted in the absence of any commercial or financial relationships that could be construed as a potential conflict of interest.
