# Supplementary material for: Monomer-Induced Customization of UV-Cured Atelocollagen Hydrogel Networks
Source: Front Chem. 2018 Dec 17;6:626. doi: 10.3389/fchem.2018.00626 (PMC6304747; doi:10.3389/fchem.2018.00626)
Supplement: Supplementary file 1 [file Data_Sheet_1.docx]

**Supporting information**

# Monomer-induced customisation of UV-cured atelocollagen hydrogel networks

He Liang,^1, 2^ Stephen J. Russell,^1^ David J. Wood,^2^ Giuseppe Tronci^1,2*^

^1^ Clothworkers’ Centre for Textile Materials Innovation for Healthcare, School of Design, University of Leeds, United Kingdom

^2^ Biomaterials and Tissue Engineering Research Group, School of Dentistry, St. James’s University Hospital, University of Leeds, United Kingdom


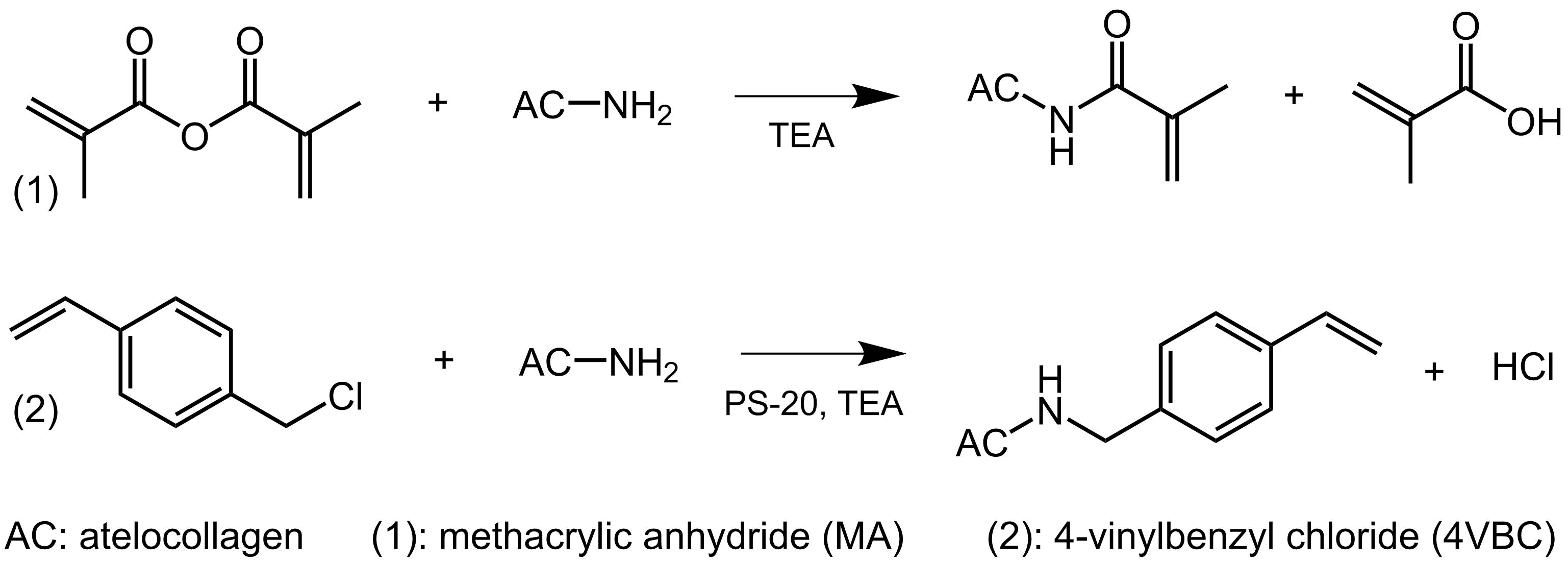


**Figure S1.** Functionalization reaction of type I atelocollagen (AC) with either MA (1) or 4VBC (2). Both reactions proceed via amine-initiated nucleophilic substitution in the presence of trimethylamine (TEA), whereby PS-20 is used to mediate the solubility of 4VBC in water.


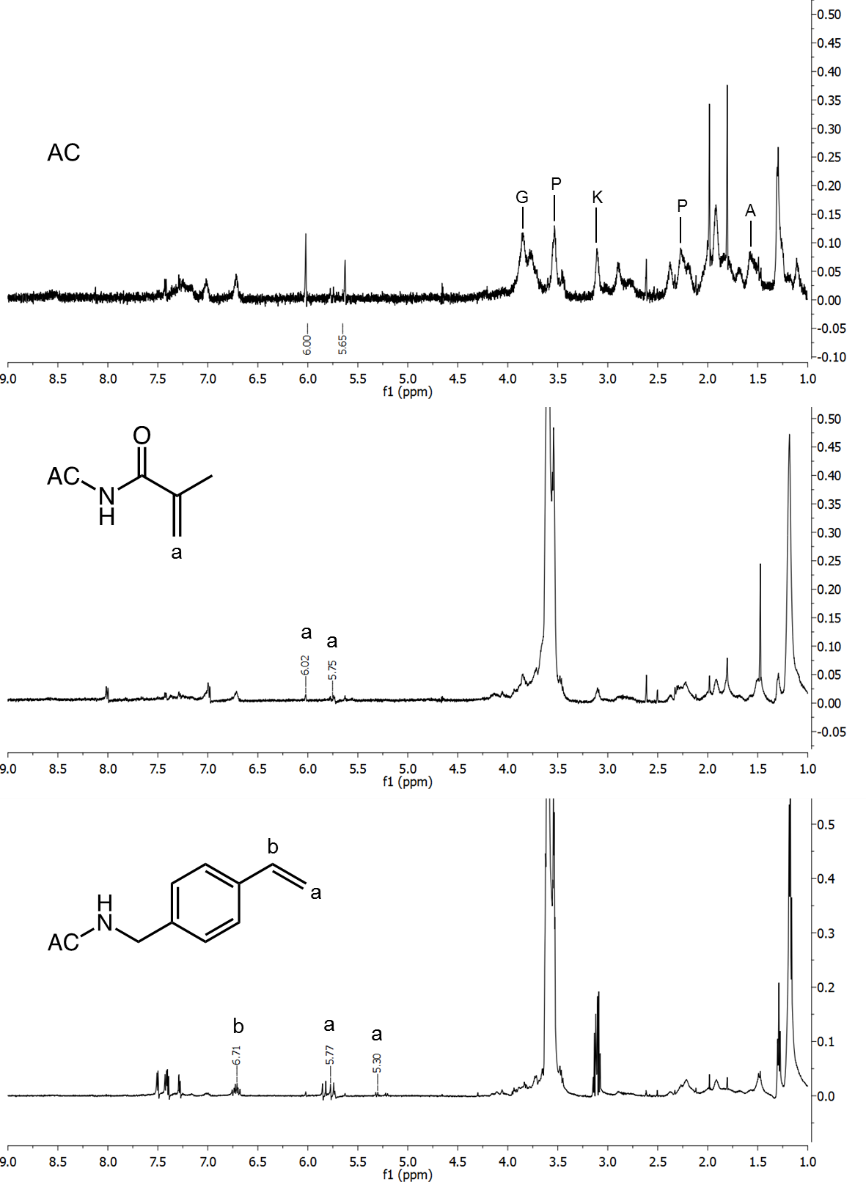


**Figure S2.** ^1^H-NMR spectra of native type I atelocollagen (AC, top), MA-functionalized AC (sample MA0.3, middle) and 4VBC-functionalized AC (sample 4VBC25, bottom), recorded in 10 mM DCl (5 mg·ml^-1^) at room temperature.

**
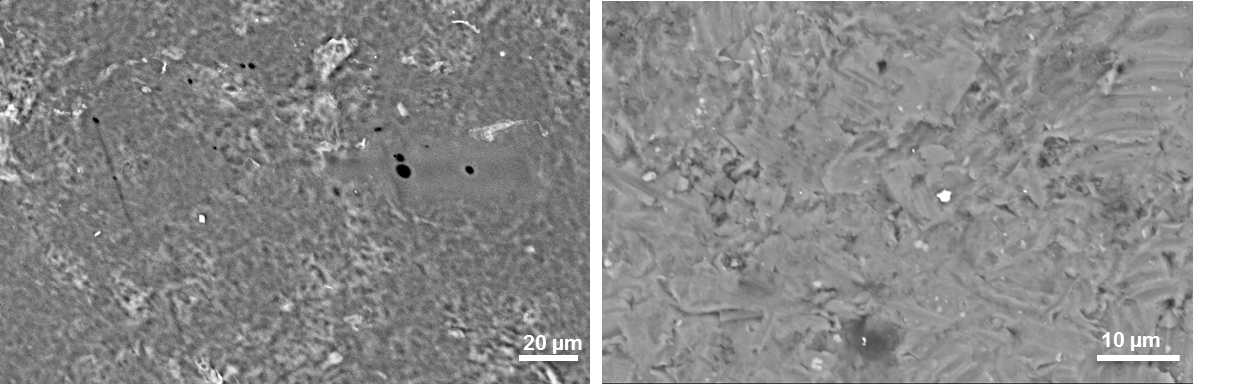
**

**Figure S3.** Typical cool-stage SEM image of G292 cell-seeded MA0.3(A)* hydrogel at day 7. No cells were found due to the effect of shrinkage.
